# [Expression of Concern] Gambogic acid inhibits invasion of osteosarcoma via upregulation of TIMP-1

**DOI:** 10.3892/ijmm.2026.6008

**Published:** 2026-10-08

**Authors:** Zeng-Feng Xin, Cheng-Chun Shen, Li-Jiang Tao, Shi-Gui Yan, Hao-Bo Wu

Int J Mol Med 31: 105-112, 2013; DOI: 10.3892/ijmm.2012.1192

Following the publication of this paper, it was drawn to the Editor's attention by a concerned reader that, regarding the cell invasion assay data shown in Figs. 2A and 5A on p. 107 and p. 100 respectively, the '0 *μ*M gambogic acid (GA)' data panel in Fig. 2A (upper left panel) contained overlapping sections of data with the 'SiRNA TIMP-1' and the 'SiRNA + GA (1 *μ*M)' data panels in Fig. 5A; furthermore, upon analyzing the data in this paper independently in the Editorial Office, it came to light that the '0 *μ*M GA' and the '0.5 *μ*M GA' data panels in Fig. 2A also contained an overlapping edge of data, suggesting that these data, which were intended to show the results from differently performed experiments, had apparently been derived from a smaller number of original sources.

The authors have been contacted by the Editorial Office to offer an explanation for the apparently incorrect assembly of this pair of figures, and we are awaiting their response. Owing to the fact that the Editorial Office has been made aware of potential issues surrounding the scientific integrity of this paper, we are issuing an Expression of Concern to notify readers of this potential problem while the Editorial Office continues to investigate this matter further.

